# Chinese Tea Alleviates CCl_4_-Induced Liver Injury through the NF-κBorNrf2Signaling Pathway in C57BL-6J Mice

**DOI:** 10.3390/nu14050972

**Published:** 2022-02-24

**Authors:** Zhaoyu Wu, Lingli Sun, Ruohong Chen, Shuai Wen, Qiuhua Li, Xingfei Lai, Zhenbiao Zhang, Fanrong Cao, Shili Sun

**Affiliations:** 1 Tea Research Institute, Guangdong Academy of Agricultural Sciences, Guangdong Key Laboratory of Tea Resources Innovation & Utilization, Guangzhou 510640, China; wuzhaoyu0323@163.com (Z.W.); lingli_318@126.com (L.S.); chenruohong@tea.gdaas.cn (R.C.); wenshuai@tea.gdaas.cn (S.W.); liqiuhua@tea.gdaas.cn (Q.L.); laixingfei@tea.gdaas.cn (X.L.); zhangzhenbiao@gdaas.cn (Z.Z.); 2 Department of Tea Science, College of Horticulture, South China Agricultural University, Guangzhou 510641, China

**Keywords:** tea, liver injury, anti-inflammation, NF-κB pathway, Nrf2 pathway

## Abstract

Liver injury is a life-threatening condition that is usually caused by excessive alcohol consumption, improperdiet, and stressful lifestyle and can even progress to liver cancer. Tea is a popular beverage with proven health benefits and is known to exert a protective effect on the liver, intestines, and stomach. In this study, we analyzed the therapeutic effects of six kinds of tea on carbon tetrachloride (CCl_4_)-induced liver injury in a mouse model. The mice were injected with 10 mL/kg 5% CCl_4_ to induce liver injury and then given oral gavage of green tea, yellow tea, oolong tea, white tea, black tea, and dark tea, respectively. The serum levels of alanine aminotransferase (ALT) and aspartate aminotransferase (AST) were measured, and the expression levels of inflammation and oxidative stress-related proteins in the liver tissues were quantified. All six kinds of tea partly reduced the liver index, restored the size of the enlarged liver in the CCl_4_ model, and decreased the serum levels of ALT and AST. Furthermore, the highly fermented dark tea significantly reduced the expression levels of NF-κB and the downstream inflammatory factors, whereas the unfermented green tea inhibited oxidative stress by activating the antioxidant Nrf2 pathway. Taken together, tea can protect against liver inflammation, and unfermented tea can improve antioxidant levels. Further studies are needed on the bioactive components of tea to develop drugs against liver injury.

## 1. Introduction

Liver injury is a multi-factorial disease caused by high alcohol intake, drugs, and chemical toxins and often progresses to cirrhosis and liver cancer [1]. Oxidative stress is the pathological basis of liver injury, fatty liver, viral hepatitis, liver fibrosis, and other liver diseases [2]. Reactive oxygen species (ROS) are invariably formed during aerobic metabolism via electron transfer across the mitochondrial respiratory chain complex. Given that the liver is rich in mitochondria, it is also highly susceptible to oxidative stress and the ensuing damage [3]. Carbon tetrachloride (CCl_4_) is routinely used to model liver injury in animal models. It induces oxidative stress in the liver cells by releasing the free radicals Cl− and CCl3− into the microsomes, which leads to lipid peroxidation, the destruction of cell membranes, the oxidation of biological macromolecules, and eventually, liver damage [4]. Oxidative stress activates the nuclear factor κ-light-chain-enhancer of the activated B cells (NF-κB) pathway [5], which culminates in the secretion of interleukin-6 (IL-6) and IL-1β, resulting in the infiltration of neutrophils and subsequent inflammatory injury [6].

Studies show that tea polyphenols can scavenge oxygen free radicals [7,8], activate antioxidant enzymes such as superoxide dismutase (SOD), glutathione (GSH), and catalase (CAT), and reduce the levels of the lipid peroxidation product malondialdehyde (MDA) [9]. In addition, tea polyphenols can prevent CCl_4_-induced lipid peroxidation and protect liver cell membranes, microsomal lipids, and proteins [10]. There is evidence that tea polyphenols can protect against peroxidation-induced jaundice and liver injury, acute liver injury caused by cadmium poisoning, alcoholic liver injury, and even liver cancer [11]. Tea is classified into green, white, yellow, oolong, black, and dark varieties depending on the extent of fermentation. Green tea and white tea are unfermented, yellow tea and oolong tea are lightly fermented, and fermented types include black tea and dark tea [12]. Fermentation significantly alters the nutrient profile of tea, and polyphenols are typically abundant in the unfermented and lightly fermented varieties. The heavily fermented dark tea, on the other hand, contains more flavonoids [13]. The various health benefits associated with tea can be attributed to polyphenols, flavonoids, caffeine, free amino acids, and soluble sugars [14,15,16]. Studies show that dark tea has a similar protective effect against liver injury togreen tea [12], and the flavonoids of black tea can reduce inflammation by downregulating inducible nitric oxide synthase (iNOS) and cyclooxygenase-2 (COX-2) [17]. Thus, we can surmise that flavonoids may also protect the liver against inflammatory damage. Most studies on the hepatoprotective effects of tea have been conducted on the Pu’er variety, although the different types produced from the same variety of tea leaves have not been compared so far.

In this study, we compared the therapeutic effects of the six types of tea processed from the Yinghong NO. 9 tea leaves on a mouse model of CCl_4_-induced liver injury and analyzed the underlying mechanisms. Our findings support further clinical investigation into the beneficial effects of tea on liver health.

## 2. Materials and Methods

### 2.1. Preparation of Tea Extracts 

Green, white, yellow, oolong, black, and dark tea leaves were obtained from the Yinghong NO. 9 cultivar planted at the Research Institute of Guangdong Academy of Agricultural Sciences, China. The tea extracts were prepared as previously described [18]. 

### 2.2. Determination of the Main Components of Tea

The water content in the tea was determined according to the Chinese national standard (GB/T 8303-2013 and GB/T 8305-2013). The content of tea polyphenols was measured using theFolin–Ciocalteu method (GB/T 83313-2018).Free amino acids were measured using the ninhydrin method (GB/T 83314-2013), and total soluble sugars were measured using the anthrone–sulfuric acid colorimetric method [19].

### 2.3. High-Performance Liquid Chromatography (HPLC) 

Standard solutions of 100μg/mL gallic acid, gallocatechin, epigallocatechin, catechin, caffeine, epicatechin, epigallocatechingallate, gallocatechingallate, epicatechingallate, and catechingallate were prepared in 50% methanol. The different tea samples were extracted with 95% methanol and filtered through 0.45μm pore size membranes. Primary catechins in the tea extracts were analyzed using the Agilent 1260 HPLC system fitted with Zorbax column (250 mm × 4.6 mm, 5μm). The samples were eluted at the flow rate of 1 mL/min. Methanol (A) and 0.1% aqueous phosphoric acid (B) were used for elution (25% B from 0 to 19.5 min). The injection volume was 5μL, and the samples were detected at 280 nm. All operations were performed at 30 °C.

### 2.4. Establishment of Murine Liver Injury Model

All animal experiments were conducted in accordance with the Animal Care and Use guidelines of Tea Research Institute Guangdong Academy of Agricultural Sciences and approved by the Institutional Animal Care and Use Committee (Serial Number: 2019003).Female C57BL/6J mice (aged eight weeks) were divided into control, model, positive control (silymarin), GT (green tea), WT (white tea), YT (yellow tea), OT (oolong tea), BT (black tea), and DT (dark tea) groups (*n* = 5 each).

The mice were acclimatized for one week, and all except the normal control group were intraperitoneally injected with CCl_4_ (10 mL/kg; CCl_4_: olive oil = 1:19, *v*/*v*) on day 1. The mice were intragastrically administered silymarin (100 mg/kg) or the respective tea extracts (100 mg/kg) daily for four days, whereas animals in the normal and model groups were given the same volume of water. After four days of treatment, the mice were fasted for 10 days and euthanized. Blood was drawn retro-orbitally, centrifuged at 2500 rpm for 20 min, and the serum was separated. The liver was removed from each mouse and weighed, and the liver index was calculated as the percentage of liver weight to body weight. All biological samples were stored at −80°C. 

### 2.5. Biochemical Assays 

Serum levels of aspartate aminotransferase (AST) and alanine aminotransferase (ALT) were determined using specific assay kits (Nanjing Jiancheng Bioengineering Institute, Nanjing, China). Here, 100 mg frozen liver samples were homogenized with 0.9 mL normal saline and centrifuged at 2500 rpm (20 min, 4 °C). The protein content in the homogenates was determined using bicinchoninic acid (BCA, Thermo, Shanghhai, China, VK312556). The levels of glutathione (GSH), malondialdehyde (MDA), catalase (CAT), and total superoxide dismutase (T-SOD) levels in the liver homogenates were measured using specific assay kits (Nanjing Jiancheng Bioengineering Institute, Nanjing, China). 

### 2.6. Histopathological Staining

Liver tissues were fixed in 10% formalin solution for 24 h, dehydrated in 75% ethanol for 24 h, and embedded in paraffin. The blocks were cut into thin sections and stained with hematoxylin and eosin (H&E) as per standard protocols. The slides were observed under a microscope (Olympus, Tokyo, Japan, 100 X), and histopathological assessment was performed as described previously [19]. 

### 2.7. Western Blotting

Liver tissue samples (100 mg) were homogenized in 990µL radio immunoprecipitation assay (RIPA) supplemented with 10 µL phenylmethanesulfonyl fluoride (PMSF). The homogenates were centrifuged at 18,506× *g* (20 min, 4 °C) to remove debris and were kept on ice for 60 min. The protein content in the lysates was measured using bicinchoninic acid (BCA, Thermo, Waltham, MA, USA, VK312556). Equal amounts of protein per sample were mixed with a quarter volume of 4× loading buffer and denatured by incubating at 98 °C in a water bath for 5 min. The proteins were separated by polyacrylamide gel electrophoresis (80–120 V) and transferred onto a polyvinylidene fluoride (PVDF) membrane. After blocking, the membranes were incubated overnight with primary antibodies (all diluted 1:1000) specific for cyclooxygenase 2 (COX-2, CST, Danvers, MA, USA, 12282S), inducible nitric oxide synthase (iNOS, SantaCruz, Dallas, TX, USA, SC-651), p-NF-κB p65 (CST, Danvers, MA, USA, 13346S), IL-1β (Bioss, MA, USA, bs-0812R), IL-6 (SantaCruz, Dallas, TX, USA, SC-1265), TNF-α (Abcam, Cambridge, UK, ab6671), HO-1 (CST, Danvers, MA, USA, 43966S), Nrf2 (CST, Danvers, MA, USA, 12721S), and β-actin (Sigma, MO, USA, A1978) at 4 °C, followed by the secondary antibody at 37 °C for 50 min. After colorimetric detection and chemiluminescence imaging, the positive bands were quantified by densitometry using Image J software. The results were normalized to the density of β-actin bands.

### 2.8. Statistical Analysis

The data are expressed as the mean ± standard error of mean (SEM). GraphPad Prism 8.0 software (GraphPad Software Inc. San Diego, CA, USA) was used for statistical analysis. The differences between the mean values for each group were assessed by one-way ANOVA with Duncan’s new multiple-range test (MRT). *p* < 0.05 was considered statistically significant. 

## 3. Results

### 3.1. Composition of the Tea Extracts

**Figure 1:**
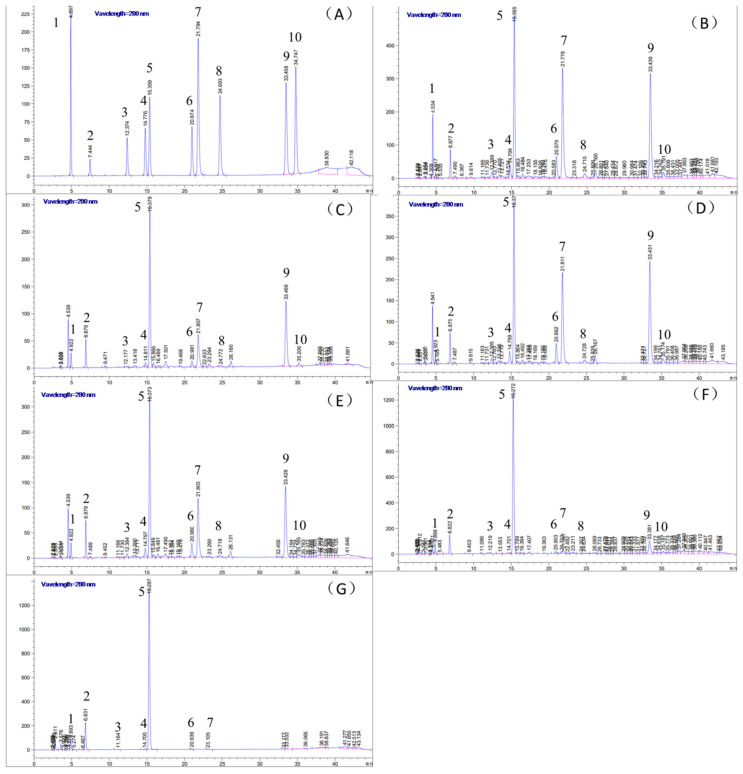
The HPLC chromatograms of the (**A**) standard compounds, (**B**) green tea, (**C**)white tea, (**D**) yellow tea, (**E**) oolong tea, (**F**) black tea and (**G**) dark teaunder 280 nm. 1, gallic acid; 2, gallocatechin; 3, epigallocatechin; 4, catechin; 5, caffeine; 6, epicatechin; 7, epigallocatechingallate; 8, gallocatechingallate; 9, epicatechingallate; 10, catechingallate.The samples were detected at 280 nm.

The main phytochemical components of the different tea extracts were identified and quantified by HPLC (Figure 1). As shown in Table 1, the green tea extracts had high levels of catechin (24.351 ± 2.161 mg/g) and epigallocatechingallate (91.779 ± 5.148 mg/g). The content of catechin gradually decreased with the degree of fermentation, indicating that the anti-inflammatory effect of dark tea may be related to the transformation of catechin. Furthermore, high levels of polyphenols were detected in green tea (27.166 ± 0.023%), whereas dark tea had the least amount (5.066 ± 0.006%). However, the highest content of flavonoids (17.694 ± 0.572%) was detected in dark tea (Table 2).

### 3.2. The Different Tea Extracts Mitigated CCl_4_-Induced Liver Injury

**Figure 2:**
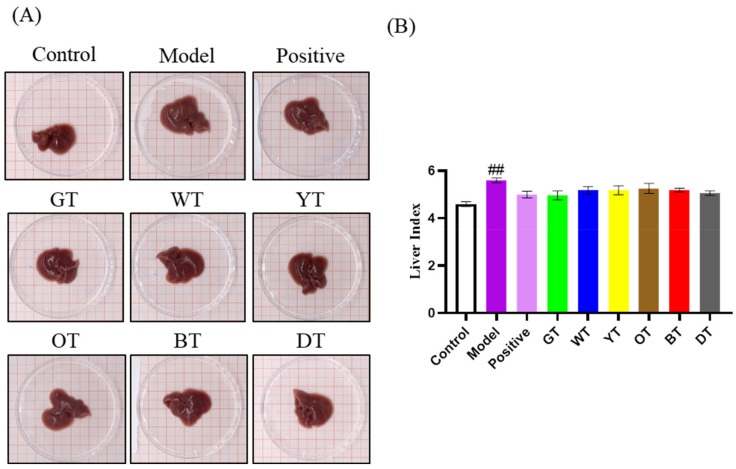
The liver (**A**) and liver index (**B**) of C57/BL mice treated with CCl_4_. D Data are expressed as mean ± SEM of at least three independent experiments (*n* ≥ 5). ^#*#*^ *p* < 0.01 Control versus Model group.

**Figure 3:**
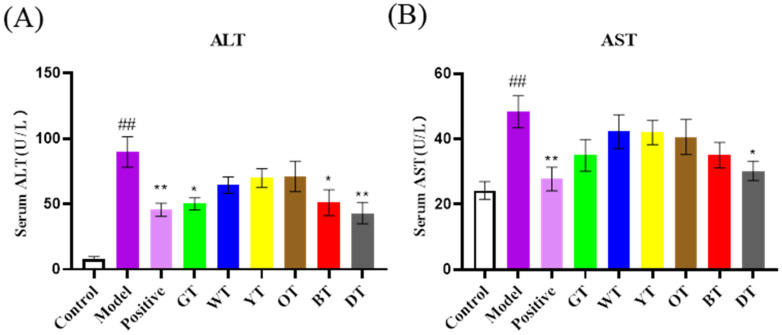
Serum levels of AST(**A**)and ALT (**B**). Data are expressed as mean ± SEM of at least three independent experiments (*n* ≥ 5). ^#*#*^ *p* < 0.01 versus control group; * *p* < 0.05 and ** p < 0.01 Tea-treated versus Model group.

As shown in Figure 2A, CCl_4_ exposure significantly enlarged the liver compared to that of the untreated control mice, which was marginally decreased by the different tea extracts. The liver index was also markedly higher in the model group compared to the control group (*p* < 0.01). The mice treated with the tea extracts showed a decrease in the liver index, although the difference was not significant (Figure 2B). Consistent with the gross observations, the serum ALT and AST levels were markedly elevated after CCl_4_ exposure, which was indicative of liver injury and dysfunction. Black tea and dark tea significantly decreased the ALT levels (*p* < 0.01; Figure 3A), and all the tea extracts, except those of oolong tea, significantly reduced the serum AST levels (*p* < 0.01; Figure 3B).

**Figure 4:**
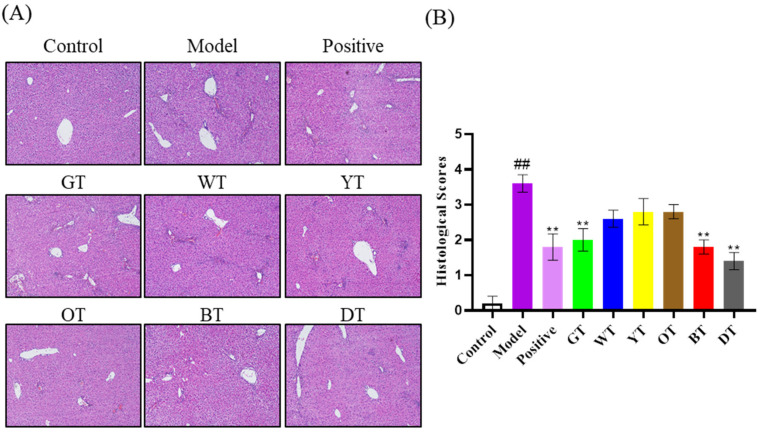
(**A**) Representative images of HE-stained liver sections from the indicated groups. (**B**) Histological scoring in the indicated groups. Normal: untreated mice; Model: CCl4-treated mice; Positive: silymarin; GT: green tea; WT: white tea; YT: yellow tea; OT: oolong tea; BT: black tea; DT: dark tea. Data are expressed as mean ± SEM of at least three independent experiments (*n ≥* 5).^#*#*^ *p* < 0.01 versus control group; ** p < 0.01 Tea-treated versus the Model group.

A histological examination of the liver revealed a round central vein, uniform hepatocytes, and radially and evenly arranged capillaries in the liver parenchyma of the control mice. CCl_4_ exposure significantly damaged the liver tissues, which was manifested as the loss of liver structure, hepatocyte necrosis, inflammatory cell infiltration in the necrotic regions, and increased intercellular gaps (Figure 4A). Black tea and dark tea supplementation significantly reduced liver injury (*p* < 0.01), restored normal cell morphology, and decreased inflammation (Figure 4B).

### 3.3. Fermented Tea Inhibited CCl4-Induced Liver Inflammation by Blocking the NF-κB Pathway

**Figure 5:**
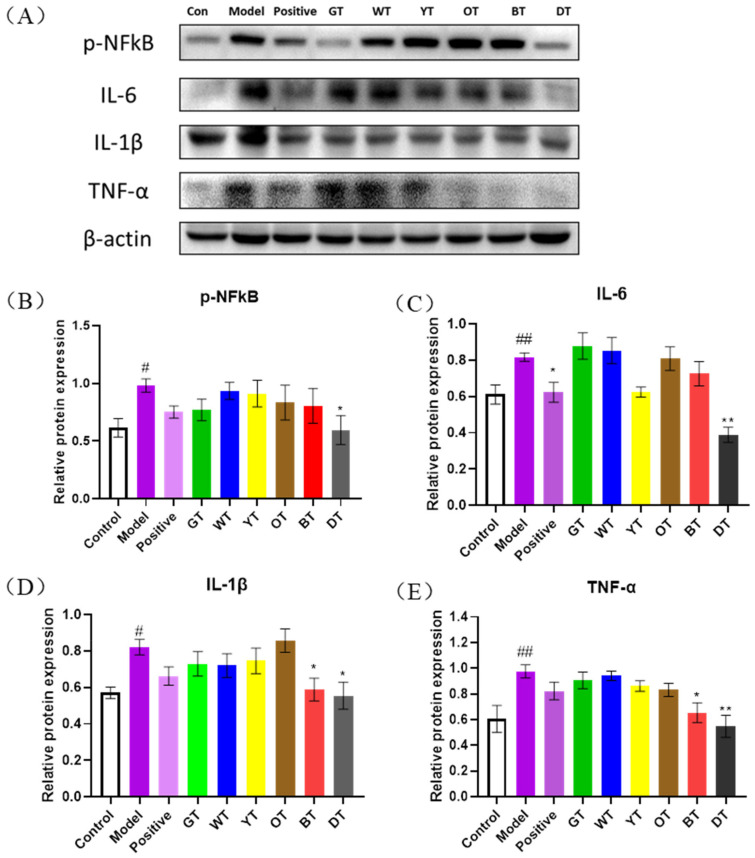
Fermented tea inhibited CCl_4_-induced inflammation by blocking the NF-κB signaling pathway. (**A**) Immunoblot showing expression levels of TNF-α, IL-1β, IL-6, and p-NF-κB and the quantification of (**B**) p-NF-κB, (**C**) IL-6, (**D**) IL-1β, and (**E**) TNF-α. β-actin was the loading control. Data are expressed as mean ± SEM of at least three independent experiments (*n ≥* 5). *^#^ p* < 0.05 and ^#*#*^ *p* < 0.01 versus control group; * p < 0.05 and ** p < 0.01 Tea-treated versus the Model group.

Since necrotic cells release copious amounts of inflammatory factors, we next analyzed the levels of various inflammatory markers in the liver tissues of the different mice in order to determine the extent of liver injury. The CCl_4_-treated mice showed a significantly higher number of cells positive for the pro-inflammatory cytokines, including IL-6, IL-1β, and TNF-α, compared to the control mice (Figure 5). These results suggest that tea can alleviate CCl_4_-induced inflammation. To further elucidate the mechanistic basis of the anti-inflammatory effects of the tea extracts, we analyzed the NF-κB pathway proteins. As shown in Figure 5, the expression levels of IL-6, TNF-α (*p* < 0.01) and IL-1β (*p* < 0.05) were significantly increased in the CCl_4_-induced model mice and decreased markedly in mice treated with dark tea and black tea extracts (*p* < 0.01, *p* < 0.05) (Figure 5C,E).Dark tea also significantly inhibited NF-κB phosphorylation (*p* < 0.05) (Figure 5B). Taken together, fermented tea varieties can alleviate CCl_4_-induced liver injury by inhibiting the NF-κB-dependent inflammatory pathway.

**Figure 6:**
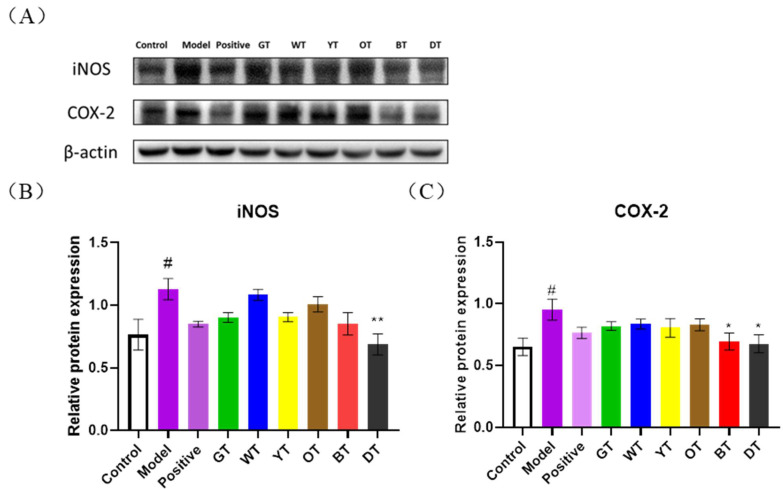
(**A**) Immunoblot showing expression levels of iNOS and COX-2 proteins in the indicated groups and the quantification of (**B**) iNOS and (**C**) COX-2. β-actin was the loading control. Data are expressed as mean ± SEM of at least three independent experiments (*n ≥* 5). *^#^ p* < 0.05 versus control group; * p < 0.05 and ** *p* < 0.01 Tea-treated versus the Model group.

iNOS and COX-2 lie downstream of the NF-κB pathway and are the two key enzymes involved in prostaglandin and NO biosynthesis, respectively. As shown in Figure 6, both iNOS and COX-2 were significantly upregulated in the model group (*p* < 0.05) and inhibited following treatment with dark tea and black tea (*p* < 0.05) (Figure 6B,C). This further proves that the protective effect of fermented tea on liver tissue is mediated through the NF-κB pathway.

### 3.4. Unfermented Tea Reduced Oxidative Stress by Activating the Nrf2 Pathway

**Figure 7:**
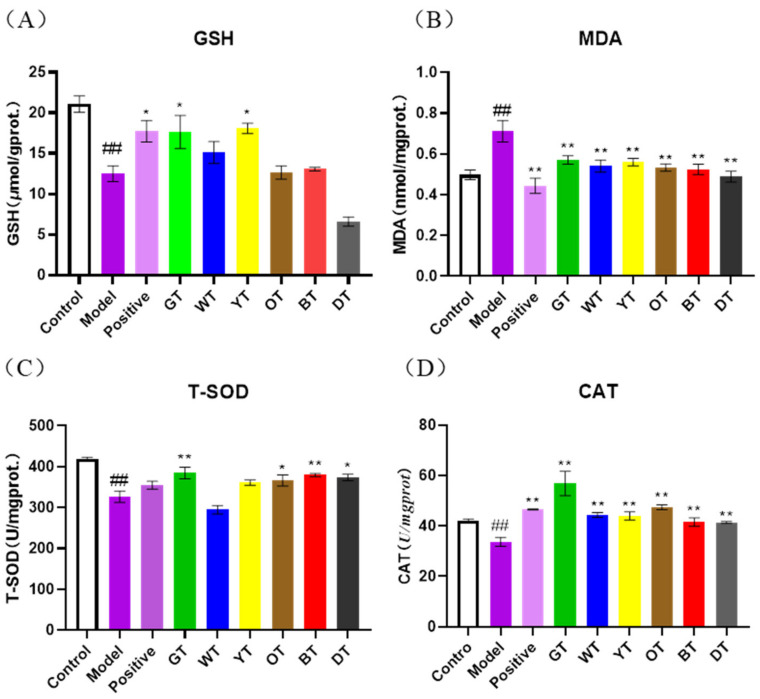
Levels of GSH (**A**), MDA (**B**), SOD (**C**), and CAT (**D**) in mice liver. Data are expressed as mean ± SEM of at least three independent experiments (*n ≥* 5). ^#*#*^ *p* < 0.01 versus control group; * p < 0.05 and ** p < 0.01 Tea-treated versus the Model group.

**Figure 8:**
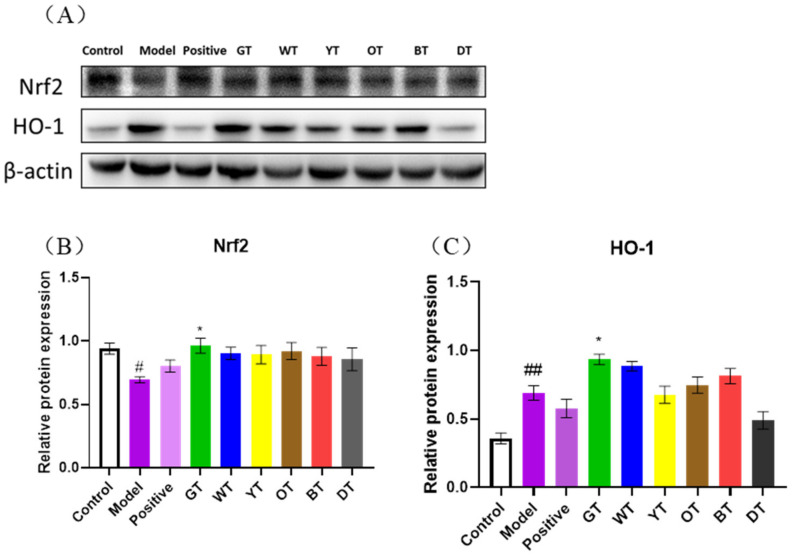
Unfermented tea enhances antioxidant activity via the Nrf2 signaling pathway in mice with CCl4-induced liver injury. (**A**) Immunoblot showing expression levels of Nrf2 and HO-1 proteins in the indicated groups, and the quantification of (**B**) Nrf2 and (**C**) HO-1. β-Actin was the loading control. Data are expressed as mean ± SEM of at least three independent experiments (*n ≥* 5). *^#^ p* < 0.05 and ^##^ *p* < 0.01versus control group; * p < 0.05 Tea-treated versus the Model group.

CCl_4_ promotes oxidative liver injury by inhibiting the antioxidant Nrf2 signaling pathway. To evaluate the antioxidant effect of the tea extracts, we analyzed the levels of GSH, MDA, SOD, and CAT in the liver tissues. As shown in Figure 7, the six types of tea significantly reduced MDA levels (*p* < 0.01) and increased those of endogenous antioxidants such as GSH, SOD, and CAT (*p* < 0.05) by varying degrees. As shown in Figure 8, Nrf2 levels were significantly lower in the CCl_4_-induced model group (*p* < 0.05; Figure 8A,B) compared to that of the controls and restored in mice treated with green tea (*p*< 0.05). CCl_4_ exposure also significantly increased the expression of HO-1 (*p* < 0.01), which was decreased following green tea administration. Taken together, fermented tea alleviates CCl_4_-induced liver injury by inhibiting the NF-κB inflammatory pathway, whereas unfermented tea inhibits hepatic oxidative stress by activating the Nrf2 signaling pathway.

## 4. Discussion

Tea is one of the three most routinely consumed non-alcoholic beverages worldwide and has proven health benefits. In this study, we found that the extracts of different types of tea obtained from the same cultivar mitigated CCl_4_-induced liver injury and dysfunction in a mouse model. Mechanistically, dark tea had a significant anti-inflammatory effect, whereas green tea alleviated the oxidative stress in the liver tissues. The content of tea polyphenols gradually decreases with the degreeof fermentation. Given that tea polyphenols are potent antioxidants and free radical scavengers, this could explain the stronger antioxidant effect of green teaas opposed to the anti-inflammatory effect of dark tea.

Acute liver injury is characterized by elevated ALT and AST in the sera. These enzymes are released from the cytoplasm (ALT) and mitochondria (AST) of damaged hepatocytes [20], and their serum levels spike significantly during hepatitis, liver trauma, and following CCl_4_-inducedacute liver injury [21], which is indicative of abnormal liver function [21,22]. In this study, we found that green tea, black tea, and dark tea inhibited aCCl_4_-induced increase in ALT and AST levels. In addition, the degree of the hepatoprotective effects of differed among the distinct varieties, which can be attributed to the difference in processing.

CCl_4_ triggers an inflammatory response in the liver, which is characterized by a significant increase in the levels of IL-6, TNF-α, and IL-1β in the serum [23]. IL-6 promotes the differentiation and proliferation of T lymphocytes, which augments inflammation. TNF-α increases the apoptosis of hepatocytes by inducing double-strand DNA breaks [24] and exacerbates liver injury by enhancing the inflammatory response through the NF-κB pathway [25]. The production of IL-1β stimulates the activation of lymphocytes, and excessive amounts can aggravate the degree of liver damage. NF-κB is the key upstream regulatory factor of the inflammatory response and increases the levels of IL-6 and TNF-α during inflammation. Under physiological conditions, NF-κB is in the inactivated state and undergoes phosphorylation in response to exogenous stimuli, thereby activating the downstream inflammatory factors [26]. iNOS is an inflammatory factor that is activated during liver injury and promotes tissue damage [27,28]. COX-2 is another inflammatory factor that is normally present at low levels and is elevated during liver injury and aggravates liver inflammation [29]. Dark tea significantly inhibited CCl_4_-induced liver injury by targeting the NF-κB inflammatory cascade and the downstream cytokines.

Studies show that CCl_4_ can cause oxidative stress by producing excessive free radicals, which eventually cause liver damage. Oxidative stress is neutralized by endogenous antioxidants such as SOD, CAT, and GSH [30]. SOD is a free radical scavenger [31] that alleviates oxidative liver damage [32,33], and its levels are significantly reduced in the liver of CCl_4_-injected mice [34]. CAT inhibits oxidative stress by quenching H_2_O_2_ and is known to reduce liver damage caused by CCl_4_ [35]. GSH also lowers the accumulation of ROS by directly binding to the free radicals, and its activity is inhibited by CCl_4_. Consistent with these findings, CCl_4_ exposure is associated with a significant increase in the quantity of the lipid peroxidation product MDA [36]. Nrf2 is a key upstream factor in the antioxidant response and up-regulates HO-1 [37], which lowers the accumulation of free radicals [38]. We found that green tea reduced oxidative damage in the affected liver by upregulating the antioxidant enzymes and activating the Nrf-2/HO-1 pathway.

**Figure 9:**
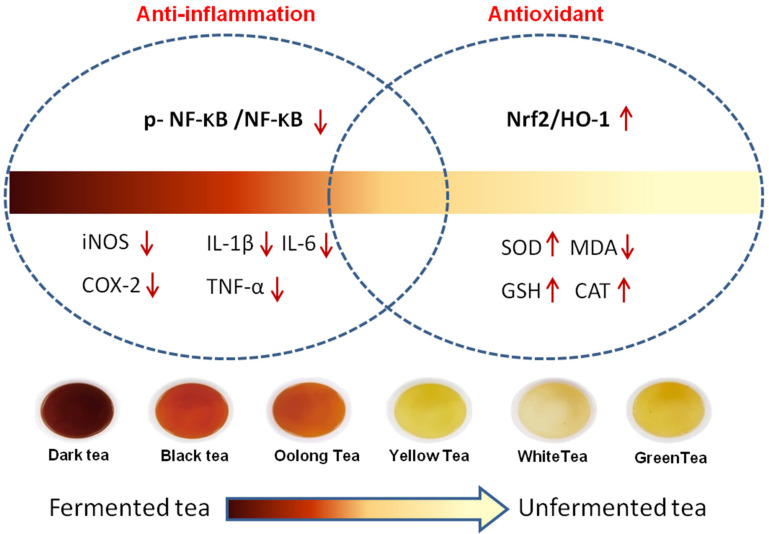
The molecular mechanisms through which the six types of tea relieve liver injury. “↑” means up-regulation of its expression level. “↓” means down-regulation of its expression level.

The different mechanisms underlying the protective effect of unfermented and fermented tea varieties is the result of fermentation-induced changes in the proportion of bioactive compounds with varying degrees of anti-inflammatory or antioxidant functions (Figure 9). To summarize, our study shows that tea extracts can protect against CCl_4_-induced acute liver injury by mitigating inflammation and reversing oxidative damage. Nevertheless, the individual bioactive components of tea need to be similarly tested, and the differences between green tea and dark tea in terms of active substances and pathways will be worth investigating.

## 5. Conclusions

We analyzed the protective effect of six kinds of tea on CCl_4_-induced acute liver injury inmice and explored the underlying mechanisms.All tea varieties mitigated CCl_4_-induced liver injury by varying degrees, decreased the liver index, and reduced serum ALT and AST levels. The protective effects of dark tea and green tea were particularly significant.The fermented dark tea mainly attenuated liver injury by inhibitingthe NF-κB pathway and the ensuing inflammatory responses, whereas the unfermented tea relieved the hepatic oxidative stress by activating theNrf2/HO-1 pathway.The distinct mechanisms are likely related to the extent of fermentation and the unique composition of bioactive compounds in the different tea varieties.In addition, microbial fermentation may lead to the transformation of certain bioactive compounds, which is a potential new research direction. Our study provides novel insights for the development of liver-protecting medicine using bioactive compounds obtained from tea.

## Figures and Tables

**Table 1:** The contents (mg/g) of main phytochemicals in six kinds of tea.

Component	GT	WT	YT	OT	BT	DT
Gallic Acid	0.370 ± 0.031 ^c^	1.220 ± 0.099 ^a^	0.803 ± 0.054 ^b^	1.342 ± 0.053 ^a^	0.956 ± 0.318 ^b^	1.310 ± 0.064 ^a^
Gallocatechin	10.628 ± 1.042 ^c^	74.952 ± 2.721 ^a^	6.606 ± 0.442 ^c^	3.432 ± 0.356 ^d^	49.921 ± 0.554 ^b^	79.080 ± 2.069 ^a^
Epigallocatechin	39.004 ± 4.015 ^a^	12.403 ± 0.795 ^c^	28.829 ± 1.240 ^b^	22.741 ± 0.756 ^bc^	7.494 ± 0.843 ^c^	1.482 ± 0.071 ^d^
Catechin	16.216 ± 0.762 ^a^	4.374 ± 0.317 ^d^	12.615 ± 1.051 ^b^	8.715 ± 0.777 ^c^	1.708 ± 0.451 ^de^	0.683 ± 0.016 ^e^
Caffeine	50.511 ± 2.229 ^a^	34.156 ± 1.425 ^b^	38.868 ± 1.983 ^b^	34.127 ± 0.115 ^b^	30.612 ± 0.509 ^c^	34.600 ± 1.023 ^b^
Epicatechin	24.351 ± 2.161 ^a^	3.607 ± 0.514 ^d^	17.523 ± 0.777 ^b^	11.605 ± 0.387 ^c^	1.922 ± 0.842 ^d^	0.570 ± 0.027 ^d^
EpigallocatechinGallate	81.779 ± 5.148 ^a^	20.714 ± 1.180 ^d^	53.648 ± 2.254 ^b^	32.914 ± 0.779 ^c^	2.266 ± 0.256 ^e^	/
GallocatechinGallate	4.990 ± 1.778 ^a^	1.554 ± 0.164 ^b^	2.817 ± 0.125 ^a^	2.240 ± 0.470 ^a^	0.808 ± 0.452 ^b^	/
EpicatechinGallate	67.266 ± 2.108 ^a^	29.543 ± 2.072 ^c^	48.878 ± 2.875 ^b^	30.301 ± 0.531 ^c^	5.788 ± 0.440 ^d^	/
CatechinGallate	0.948 ± 0.233 ^b^	1.330 ± 0.200 ^ab^	2.417 ± 0.507 ^a^	0.584 ± 0.339 ^b^	0.875 ± 0.153 ^b^	/

Values represent means ± SD (*n* =3). Different letters (a, b, c, d) in the same row indicate significant differences between mean values (*p* < 0.05).

**Table 2:** Main components of six kinds of tea.

Component	GT	WT	YT	OT	BT	DT
Water (%)	4.675 ± 0.013 ^a^	7.775 ± 0.017 ^a^	4.050 ± 0.025 ^a^	5.000 ± 0.015 ^a^	6.100 ± 0.022 ^a^	8.400 ± 0.012 ^a^
Water Extract(%)	42.413 ± 0.022 ^b^	52.377 ± 0.004 ^a^	43.527 ± 0.019 ^b^	43.692 ± 0.008 ^b^	55.326 ± 0.012 ^a^	47.891 ± 0.018 ^ab^
Tea Polyphenols(%)	27.166 ± 0.023 ^a^	16.394 ± 0.007 ^bc^	20.705 ± 0.00 ^b^	16.261 ± 0.014 ^bc^	9.774 ± 0.006 ^c^	5.066 ± 0.006 ^cd^
Amino Acid(%)	2.798 ± 0.291 ^a^	2.843 ± 0.051 ^a^	3.051 ± 0.189 ^a^	3.247 ± 0.094 ^a^	3.207 ± 0.077 ^a^	1.446 ± 0.054 ^b^
Flavonoid(%)	5.636 ± 1.267 ^bc^	9.888 ± 0.262 ^b^	6.530 ± 0.136 ^b^	5.261 ± 0.270 ^c^	16.228 ± 0.087 ^a^	17.694 ± 0.572 ^a^
Soluble Sugar(%)	6.644 ± 0.003 ^c^	11.486 ± 0.002 ^a^	8.231 ± 0.000 ^b^	8.840 ± 0.003 ^b^	6.056 ± 0.003 ^c^	7.103 ± 0.002 ^bc^

Values represent means ± SD (*n* = 3). Different letters (a, b, c, d) in the same row indicate significant differences between mean values (*p* < 0.05).

## Data Availability

The data presented in this study are available on request from the corresponding author. The data are not publicly available due to privacy reasons.
